# Assessing fatigue and sleep in chronic diseases using physiological signals from wearables: A pilot study

**DOI:** 10.3389/fphys.2022.968185

**Published:** 2022-11-14

**Authors:** Emmi Antikainen, Haneen Njoum, Jennifer Kudelka, Diogo Branco, Rana Zia Ur Rehman, Victoria Macrae, Kristen Davies, Hanna Hildesheim, Kirsten Emmert, Ralf Reilmann, C. Janneke van der Woude, Walter Maetzler, Wan-Fai Ng, Patricio O’Donnell, Geert Van Gassen, Frédéric Baribaud, Ioannis Pandis, Nikolay V. Manyakov, Mark van Gils, Teemu Ahmaniemi, Meenakshi Chatterjee

**Affiliations:** ^1^ VTT Technical Research Centre of Finland Ltd., Tampere, Finland; ^2^ Sanofi R&D, Frankfurt, Germany; ^3^ Department of Neurology, University Hospital Schleswig-Holstein, Kiel University, Kiel, Germany; ^4^ LASIGE, Faculdade de Ciências, Universidade de Lisboa, Lisbon, Portugal; ^5^ Translational and Clinical Research Institute, Faculty of Medical Sciences, Newcastle University, Newcastle Upon Tyne, United Kingdom; ^6^ NIHR Newcastle Biomedical Research Centre and NIHR Newcastle Clinical Research Facility, Newcastle Upon Tyne Hospitals NHS Foundation Trust, Newcastle Upon Tyne, United Kingdom; ^7^ George-Huntington-Institute, University of Münster, Münster, Germany; ^8^ Department of Clinical Radiology, University of Münster, Münster, Germany; ^9^ Department of Neurodegenerative Diseases and Hertie Institute for Clinical Brain Research, University of Tübingen, Tübingen, Germany; ^10^ Department of Gastroenterology and Hepatology, Erasmus MC, Rotterdam, Netherlands; ^11^ Department of Psychiatry, Harvard Medical School, McLean Hospital, Belmont, MA, United States; ^12^ Takeda Belgium, Zavente, Belgium; ^13^ Bristol Myers Squibb, New York, NY, United States; ^14^ Janssen Research & Development, London, United Kingdom; ^15^ Janssen Research & Development, Beerse, Belgium; ^16^ Faculty of Medicine and Health Technology, Tampere University, Tampere, Finland; ^17^ Janssen Research & Development, Cambridge, MA, United States

**Keywords:** wearabe sensors, chronic disease, biomedical signal analysis, fatigue, sleep disturbance, continuous monitoring, neurodegenerative diseases, immune-mediated inflammatory disease

## Abstract

Problems with fatigue and sleep are highly prevalent in patients with chronic diseases and often rated among the most disabling symptoms, impairing their activities of daily living and the health-related quality of life (HRQoL). Currently, they are evaluated primarily *via* Patient Reported Outcomes (PROs), which can suffer from recall biases and have limited sensitivity to temporal variations. Objective measurements from wearable sensors allow to reliably quantify disease state, changes in the HRQoL, and evaluate therapeutic outcomes. This work investigates the feasibility of capturing continuous physiological signals from an electrocardiography-based wearable device for remote monitoring of fatigue and sleep and quantifies the relationship of objective digital measures to self-reported fatigue and sleep disturbances. 136 individuals were followed for a total of 1,297 recording days in a longitudinal multi-site study conducted in free-living settings and registered with the German Clinical Trial Registry (DRKS00021693). Participants comprised healthy individuals (*N* = 39) and patients with neurodegenerative disorders (NDD, *N* = 31) and immune mediated inflammatory diseases (IMID, *N* = 66). Objective physiological measures correlated with fatigue and sleep PROs, while demonstrating reasonable signal quality. Furthermore, analysis of heart rate recovery estimated during activities of daily living showed significant differences between healthy and patient groups. This work underscores the promise and sensitivity of novel digital measures from multimodal sensor time-series to differentiate chronic patients from healthy individuals and monitor their HRQoL. The presented work provides clinicians with realistic insights of continuous at home patient monitoring and its practical value in quantitative assessment of fatigue and sleep, an area of unmet need.

## 1 Introduction

Health-related quality of life (HRQoL) and ability to conduct activities of daily living (ADL) are greatly impaired in patients with chronic diseases, such as neurodegenerative disorders (NDD) and immune mediated inflammatory diseases (IMID) (Kluger et al., 2013; Zielinski et al., 2019). Fatigue and sleep disturbances are known to be key factors predicting poor HRQoL or reduced ADLs, and as such alleviation of these symptoms may significantly improve patient’s health and quality of life (Center for Disease Control and Prevention, 2000). Current evaluations rely primarily on patient reported outcomes (PROs) which are subjective and prone to recall biases and poorly capture variability over time (Stone et al., 2002). Sensors, such as wearable technology or standalone sensors using a wide range of technologies, can perform continuous real-world monitoring of patient health and thus offer the opportunity to provide digital measures that are objective, potentially reliable and more sensitive to change over time (Bangerter et al., 2020a; 2020b; Luo et al., 2020).

Fatigue is defined as a multi-dimensional phenomenon in which the biophysiological, cognitive, motivational and emotional state of the body is affected resulting in significant impairment of the individual’s ability to function in their normal capacity (Davies et al., 2021). Specifically, in NDD and IMID patients, such as those with Huntington’s Disease (HD), Parkinson’s Disease (PD), Inflammatory Bowel Diseases (IBD), Primary Sjögren’s Syndrome (PSS), Rheumatoid Arthritis (RA), and Systemic Lupus Erythematosus (SLE), fatigue and sleep disturbances are highly prevalent (Hewlett et al., 2011; Lendrem et al., 2014; Siciliano et al., 2018; Chavarría et al., 2019). Previous studies assessing fatigue through digital measurement technologies are relatively sparse, especially in chronic disease populations. Changes in physical activity levels such as daily and bouted moderate to vigorous physical activity (MVPA) minutes and no bouts of MVPA have been found to be associated with fatigue in RA, SLE and Crohn’s disease (Legge et al., 2017). Fatigue has also been shown to be correlated with changes in the frequency spectrum of EEG signals (Zhang et al., 2020). Individuals with chronic fatigue syndrome were found to have lower heart rate variability (HRV) measures such as standard deviation of the interbeat intervals of normal sinus beats (SDNN), power spectrum densities of low frequency (LF) and high frequency (HF) compared to controls, while total HRV power within the frequency range of 0–0.4 Hz was shown to be negatively associated with fatigue (Boissoneault et al., 2019; Escorihuela et al., 2020).

Sleep disorders such as decreased sleep efficiency and increased fragmentation are the second most frequent complaint in PD (Stefani and Högl, 2019). In HD, sleep and circadian rhythm alterations have been reported to correlate with depression and cognitive impairment (Aziz et al., 2010). Sleep disturbances, also common in RA, SLE, IBD, and PSS, have been attributable to changes in circadian rhythms or disease symptoms such as pain, discomfort, respiratory and movement disorders sleep, with disruptions in sleep associated with further worsening of disease symptoms (Swanson and Burgess, 2017). Recording night ECG allows evaluation of the fluctuation of the sympathetic and parasympathetic nervous system functions, which physiologically happen during sleep. The LF (frequency range 0.04–0.15 Hz) reflects both sympathetic and vagal modulations, which decrease with the depth of sleep. The HF (frequency range 0.15–0.4 Hz) is associated with respiration and reflects the activity of the parasympathetic nervous system, which increases in deep sleep (Somers et al., 1993).

Digital measures that can objectively assess HRQoL-related factors, such as sleep and fatigue will be invaluable for drug development. Despite the advent of wearable sensors, there is limited understanding of fatigue and sleep assessment using objective measurements in these patient population, with existing work primarily focusing on the relationship between physical activity measured from accelerometers with fatigue PROs. Even among healthy cohorts, only few recent studies have utilized wearable sensors (Luo et al., 2020) such as inertial measurement units and heart rate monitors to assess fatigue, majority with smaller sample size or under tightly controlled experimental settings. Building on these challenges, the IDEA-FAST project (https://idea-fast.eu/) aims to utilize multiple sensing modalities and technologies at home to identify digital endpoints of fatigue and sleep in the six NDD and IMID populations–HD, PD, IBD, PSS, RA, and SLE.

In this paper, we present insights from a feasibility study of IDEA-FAST (The IDEA-FAST project consortium, 2020) and focus specifically on evaluating the promise of capturing digital measures of fatigue and sleep from biophysiological signals collected in patients and healthy groups at home from a wearable ECG device. Specifically, signal quality and coverage of digital measures were assessed and their agreement with sleep and fatigue PROs were investigated. Furthermore, heart rate recovery (HRR) periods were estimated, among patients and healthy participants, as a metric to assess physiological fitness which could potentially be impacted by fatigue. Post-exercise heart rate recovery reflects the interplay between the sympathetic and parasympathetics parts of the autonomic nervous system (Qiu et al., 2017). It is an important predictor of all-cause mortality and related to fatal cardiovascular events (Qiu et al., 2017). Decrease in HRR is shown to be associated with physical fatigue (Lamberts et al., 2009; Djaoui et al., 2017) and has been typically measured in controlled laboratory settings. Here we explored if HRR quantified from free-living environments can distinguish between NDD, IMID and healthy groups and those with varying levels of fatigue.

## 2 Materials and methods

The presented data was obtained as a part of the IDEA-FAST project (The IDEA-FAST project consortium, 2020; Chen et al., 2022). Nine different candidate technologies measuring different modalities (activity trackers, ECG-sensors, sleep trackers) were explored in a feasibility study aiming to assess fatigue and sleep disorders. Additionally, the participants’ social activity, cognitive skills, and PROs were captured with smartphone applications. This paper focuses on the continuously measured physiological signals collected from the ECG-based VitalPatch sensor and the concurrently collected PROs. The digital measures from VitalPatch included heart rate (HR), R-to-R interval, respiratory rate (RR), skin temperature (skin T), number of steps, and posture. The first three are mainly derived from the ECG measurement and are the main focus of this study.

### 2.1 Ethical approvals

Ethical approval was first granted by the Ethical Committee of the Medical Faculty of Kiel University (D491/20) in June 2020 and then by the Research Ethics Committees of all other study sites: Newcastle upon Tyne Hospitals National Health Service (NHS) Foundation Trust/Newcastle University in August 2020, Erasmus University Medical Centre in Rotterdam in November 2020, and George-Huntington-Institute in Münster in September 2020. The study was registered with the German Clinical Trial Registry (DRKS00021693) and was conducted according to the principles of the Declaration of Helsinki (version of 2013).

### 2.2 Study participants

Potential participants were identified during routine clinical visits at the hospitals and through public outreach at information events or support groups. After providing information about the study and obtaining informed consent, the participants were screened for eligibility. Inclusion criteria required age over 18 years, consent to participate in the study for up to 60 days and according to the study protocol, use of a smartphone in the past 3 months, and ability to follow written and oral instructions in the local language, to walk, sit, and stand independently and to socialize and communicate. Another inclusion criterion was a score of over 15 points in the Montreal Cognitive Assessment (MoCA), which was used to evaluate cognitive abilities (Nasreddine et al., 2005). Participants were excluded if they had certain comorbidities like major sleep disorders, chronic fatigue syndrome, respiratory, cardiovascular or metabolic disorders or physical traumas with hospitalization in the past 3 months, diagnosis of cancer in the past 3 years, major psychiatric disorders, suicidal attempt in the past 5 years or suicidal ideation in the past 6 months, substance or ethanol abuse or severe visual impairment.

The study was conducted at four different sites: Rotterdam (E), Kiel (K), Muenster (G), and Newcastle (N). The study start date was between July 2020 (Kiel) and November 2020 (Rotterdam), depending on the date of ethical approval of the study site. The last visit of the final participant took place in December 2021. The participants were either healthy or suffered from one of six diseases, which we have divided into two groups: NDDs including HD and PD, and IMIDs including IBD, PSS, RA, and SLE. Thus, the study inspects three participant categories: 1) the healthy participants, 2) the NDD patients, and 3) the IMID patients.

### 2.3 Study design

Participants were enrolled in the study for up to 60 days. Demographic information was collected during a baseline visit conducted at the study center or at the participant’s home. Subsequently, the participants were provided with a detailed explanation of the devices and the applications. In addition, they received informational materials and telephone support by the study team. Optional home visits were conducted to further ensure accurate use of the devices.

**FIGURE 1:**
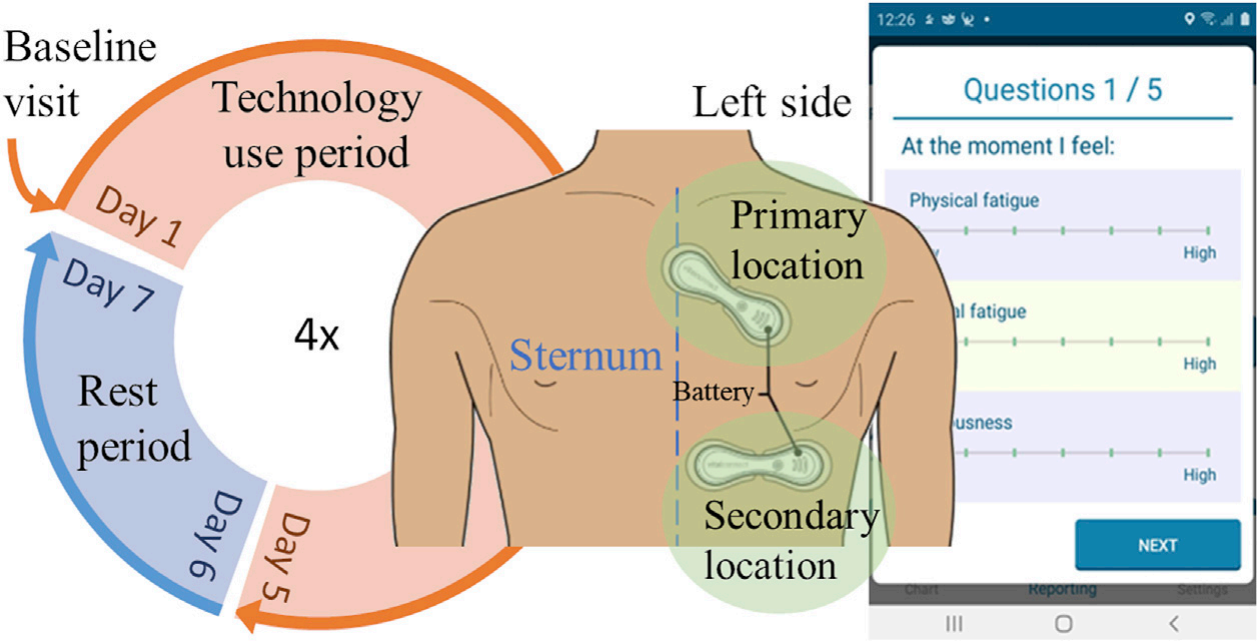
The study cycle. Participants went through a baseline visit at the beginning of the study, followed by four technology use periods (the circle) when they were instructed to use different sensors and report certain outcomes with a mobile phone application (example screenshot on the right). This study focuses on the participants using the depicted wearable device, VitalPatch (two alternative wear locations depicted).

Over a period of five consecutive days, participants wore the VitalPatch biosensor in their home environment and were instructed to carry out their usual daily activities. This constituted one technology use period that was followed by at least two rest days, after which a new technology use period could be started. Participants were able to opt for a prolonged resting period. The study cycle, illustrated in Figure 1, was repeated up to four times per participant. During the technology use period, participants were asked to report their perceptions of fatigue and sleep quality four times daily in an e-diary using the VTT Stress Monitor Application (SMA) (Vildjiounaite et al., 2018).

### 2.4 Measurement setup

VitalPatch is a wireless wearable patch sensor designed for remote patient monitoring (Areia et al., 2021). The fully disposable 12-cm patch adheres to the skin and is worn on the left chest. It contains a zinc-air battery that lasts up to 7 days. Once the measurement is started, it continues whenever the device is in skin contact, until the battery runs out. After one patch sensor is disposed, the measurement can be continued with a new patch. VitalPatch has CE certification as a Class IIa medical device and FDA clearance.

The VitalPatch biosensor incorporates a single-lead ECG, a tri-axial accelerometer, and a thermistor. It records ECG at 125 Hz sampling frequency, with derived heart rate, R-to-R interval, and respiratory rate (partly derived from the accelerometer) sampled at 0.25 Hz. The accelerometer is used for step counting and posture detection at 1 Hz. The thermistor collects skin temperature at 0.25 Hz. The recorded data is encrypted and transferred with a latency in the order of seconds *via* a wireless connection to a cloud-based patient monitoring platform. If the connection is interrupted, the device can store up to 10 h of data until the connection is re-established.

### 2.5 Patient reported outcomes

PROs were collected using the VTT Stress Monitor Application (SMA), an Android smartphone application that provides a user interface for questionnaires (Vildjiounaite et al., 2018). PROs were collected four times a day (at 9:00, 13:00, 17:00, and 21:00 local time). The response could be submitted within 3 hours of the prompted question, except in the evening as those responses were set due at 23:30. To promote compliance, the application prompted a new notification again every 15 min if the user had opened the application but did not submit the responses, and the application had gone out of active view. Throughout the day, the participants were requested to respond to a total of 14 different PROs, as detailed in Table 1. All Likert items had seven options from low (zero) to high (six). An example of the Likert item interface is presented in Figure 1.

**TABLE 1:** Patient reported outcomes collected with the VTT Stress Monitor Application.

		Questionnaire time			
Patient reported outcome	Type	Morning (9–12)	Early after-noon (13–16)	Late after-noon (17–20)	Evening (21–23:30)
Physical fatigue	Likert item	X	X	X	X
Mental fatigue	Likert item	X	X	X	X
Anxiousness	Likert item	X	X	X	X
Depression	Likert item	X	X	X	X
Pain	Likert item	X	X	X	X
I went to bed at	Clock	X			
I woke up	Clock	X			
How was your sleep?	Likert item	X			
Time to fall asleep	Drop-down menu	X			
Time awake during night	Drop-down menu	X			
Sleepiness, current feeling	Drop-down menu		X	X	X
My activities of the day, physically	Likert item				X
My activities of the day, mentally	Likert item				X
Other comments	Free text				X

### 2.6 Data pre-processing

The HR, R-to-R interval, RR, and skin T data were pre-processed in two steps. First, timestamps were sorted, and duplicates removed. Second, the data were cleaned from 1) invalid values (unsuccessfully measured) predefined by the manufacturer, 2) physiologically unrealistic values, and 3) contextual outliers. Such values were removed and considered gaps in the data, except for cases 2–3 for R-to-R interval, which were replaced using linear interpolation to improve heart rate variability (HRV) analysis (detailed below). The first pre-processing step along with the removal of invalid values were also applied to the number of steps and posture.

To exclude any physiologically unrealistic values, a range of acceptable values was defined for each feature independently. The selected limits are presented in Table 2. The limits for HR and R-to-R interval are adopted from previous studies (Tanaka et al., 2001; Zhai et al., 2020). The range for respiratory rate, on the other hand, was defined broadly, including abnormal hypo-and hyperventilation scenarios, such as exercise (Cretikos et al., 2008; Gutierrez et al., 2016; Nicolò et al., 2017, 2020). Finally, skin temperature is presumed to obtain lower values as compared to core body temperature but is allowed a range that can capture abnormal physiological states (Martinez-Nicolas et al., 2015; Rajbhandary and Nallathambi, 2020). Restricting the range is expected to exclude notably exceptional measurement conditions, even though the thermal sensor itself has been reported to work accurately across a wider range (Selvaraj et al., 2018).

**TABLE 2:** Accepted range for each physiological feature. The selected ranges were validated visually and by comparing them against the 1st and 99th percentiles of the collected data.

	Heart rate (bpm)	R-to-R interval (ms)	Respiratory rate (bpm)	Skin temperature (°C)
Minimum	30	300	4	28
Maximum	200	2000	60	40

Contextual outliers were removed to reduce unlikely variation within short time periods. Using a sliding window, the value at the centre of the window was inspected: if it was not within a predefined range of the window mean, it was considered a contextual outlier (Karlsson et al., 2012). The ranges were 30% for all features except for RR; threshold for respiration rate was 50%. As respiration can be controlled at will, it is more prone to larger variations. The size of the sliding window was 1 min for HR and R-to-R interval, 3 min for RR, and 5 min for skin T.

Patient reported outcomes did not require pre-processing, apart from the sleep times: they were collected with a 24-h clock user interface, which was discovered prone to 12-h shifts in the user input, especially when reporting late hours (12–24). Bedtimes that exceeded the waking up or occurred considerably late with respect to the wake-up time, were considered as input errors and shifted by 12 h.

### 2.7 Data quality assessment

The quality of the digital measures was assessed *via* the extent of pre-processing necessary [corresponding to items (1), (2), (3) as described in Section 2.6], and the data coverage after pre-processing. For HR, RR and skin T, coverage was calculated based on the expected number of samples. Coverage of R-to-R interval was estimated *via* the sum of recorded R-to-R interval values divided by the duration of the actual measurement period. Data quality was first evaluated on participant-level and then averaged over cohorts or participant groups. The participant-level coverage was computed as the mean of midnight-to-midnight coverage values.

Additionally, PRO coverage during VitalPatch wear periods was evaluated for each PRO as compared to the expected number of responses. PRO coverage was also evaluated midnight-to-midnight for each participant and then averaged over participant subgroups.

### 2.8 Feature aggregates

The features were segmented into time windows of interest (see Section 2.9) and aggregated into statistical descriptors, to summarize the physiological feature time series into single values, which could be compared to the corresponding PROs. The selected statistical aggregations were the mean, standard deviation (SD), minimum, and maximum.

Additionally, HRV parameters were computed from the R-to-R interval data over each full window. Furthermore, the feature coverage within each window was computed for reliability evaluation. For HRV analysis, the R-to-R interval data was further cleaned to achieve normal-to-normal (NN) intervals by replacing ectopic peaks using linear interpolation (Peltola, 2012). This was performed *via* the Malik method: intervals deviating more than 20% from the previous interval were replaced (Malik et al., 1996). Both time and frequency domain HRV features were computed, as well as geometric and non-linear features (Malik et al., 1996; Champseix, 2021). The included HRV features are described in Table 3. Details of these widely-used HRV parameters and their implications in health and performance can be found in existing literature (Shaffer and Ginsberg, 2017).

**TABLE 3:** Heart rate and heart rate variability features.

Abbreviation	Domain	Description
NN mean	Time	Mean of normal-to-normal peak intervals (NN)
NN CV	Time	Coefficient of variation of NN
NN SD	Time	Standard deviation of NN
NN median	Time	Median of NN
NN range	Time	Difference between maximum and minimum of NN
RMSSD	Time	Root mean square of consecutive differences in adjacent NN
CVSD	Time	Coefficient of variation of consecutive differences in adjacent NN
SDSD	Time	Standard deviation of consecutive differences in adjacent NN
NN50	Time	Number of interval differences greater than 50 ms
NN20	Time	Number of interval differences greater than 20 ms
pNN50	Time	Percentage of interval differences greater than 50 ms
pNN20	Time	Percentage of interval differences greater than 20 ms
HRV HR mean	Time	Heart rate mean
HRV HR SD	Time	Heart rate standard deviation
HRV HR min	Time	Heart rate minimum
HRV HR max	Time	Heart rate maximum
VLF	Frequency	Power spectral density in very low frequencies (0.003–0.04 Hz)
LF	Frequency	Power spectral density in low frequencies (0.04–0.15 Hz)
HF	Frequency	Power spectral density in high frequencies (0.15–0.40 Hz)
Total power	Frequency	Total power spectral density; sum of VLF, LF, and HF
LF/HF	Frequency	The ratio of LF and HF
LFnu	Frequency	LF normalized to the sum of LF and HF
HFnu	Frequency	HF normalized to the sum of LF and HF
Triangular index	Geometrical	Number of all NN divided by the maximum of the NN density distribution
CSI	Non-linear	Cardiac sympathetic index
mCSI	Non-linear	Modified cardiac sympathetic index
CVI	Non-linear	Cardiac vagal index
SD1	Non-linear	Poincaré plot, SD1
SD2	Non-linear	Poincaré plot, SD2
SD2/SD1	Non-linear	SD2 to SD1 ratio

### 2.9 Time windows of interest

The feature aggregates were computed over 2-h windows preceding the time at which a PRO response was obtained. Thus, the aggregation represents the participants’ physiological features leading to the questionnaire response.

To estimate physiological measures during rest, major rest periods were identified for each participant, using step count and posture information available from VitalPatch. As a proxy for major rest periods, the L5 metric was calculated which corresponded to the least active 5-h (L5) periods in the day (Witting et al., 1990). A maximum of 100 steps was allowed and a minimum of 80% laying down was used as a threshold. The starting times of the 5 h long resting windows were located at 1 min resolution, and the best option among overlapping consecutive windows was selected by maximizing the laying down percentage.

### 2.10 Feature normalization

Physiological parameters are affected by the subject’s age and sex (and physical fitness) and the inter-individual differences can be significant (Voss et al., 2015; Garavaglia et al., 2021). Therefore, the 2-h feature aggregates were normalized on a subject-by-subject basis to alleviate the differences. Previous studies have normalized HRV features by adjusting the feature according to feature baseline and range, adjusted with the 5th and 95th percentiles to account for outlier effects (Wijsman et al., 2011; Xiao et al., 2013; Altini et al., 2014; Altini and Kinnunen, 2021). In this study, the features are normalized relative to the L5 aggregates, according to

$$x_{norm}=\frac{x-{µ}_{L5}}{\sigma_{L5}},$$

where x is a feature aggregate (over a 2h window of interest), $x_{norm}$ is the normalized feature aggregate, ${µ}_{L5}$ is the mean feature value and $\sigma_{L5}$ its standard deviation obtained as the mean and SD (a) from the nearest previous L5 window, or (b) averaged over all subject specific L5 windows. In approach (a), the specific instance of x was excluded if no previous L5 window existed. Normalization was applied to all physiological feature aggregates (excluding the feature coverage).

### 2.11 Feature association with patient reported outcomes

The association between the above-described 2-h feature aggregates and the PROs were studied through repeated measures correlation, to account for intra-individual dependencies in the data (Bakdash and Marusich, 2017). Significance level α was set to 0.05. Feature aggregates demonstrating lower than 70% coverage over the window of interest were excluded from the association analysis. Moreover, only participants with at least three pairs of PROs and feature aggregates were included. Repeated measures correlation values close to one indicate linear correlation between the two compared measures.

### 2.12 Heart rate recovery

Heart rate recovery (HRR) was defined as the maximum difference in the HR signal provided by the VitalPatch sensor that was observed during a 1 min resting period after a 6-min walk, similarly to a six-minute walking test (Roberts et al., 2006; Bellet et al., 2012). Because the measurements were conducted in free-living settings, applicable sequences were retrospectively detected from the clean (non-aggregated) sensor data. The walking periods were identified *via* the “walking” posture, as classified by the wearable sensor. A walk was required to last at least 6 min, but no upper limit was applied. Small pauses in walking and changes of posture lasting up to 3 s were ignored (Del Din et al., 2016). However, a minimum average cadence of 60 steps/min was required (Sokas et al., 2021). For the 1-min resting periods, we required 100% heart rate coverage and zero taken steps. In case of multiple applicable sequences, the highest HRR for a participant was selected as the representative value.

### 2.13 Statistical analysis

One-way and two-sided Analysis of Covariance (ANCOVA) was used to assess whether the HRR differs significantly among the three participant groups (healthy, NDD, and IMID). The significance level α was again 0.05. Age and gender were taken as covariates and the effect size was evaluated using partial η^2^ (eta squared). Pairwise differences between the groups were analysed in post hoc tests performed with Tukey’s method, which adjusts the *p*-values for multiple comparisons.

All presented boxplots depict the median as the horizontal line within the box, the interquartile range (IQR) *via* the box limits, and 1.5 times the IQR through the whiskers (points falling outside this range are displayed individually as outliers).

## 3 Results

### 3.1 Participant number and demographics

Continuous physiological monitoring of VitalPatch was conducted on 136 participants, 101 of which responded to PROs collected during the patch measurement period. Participants recorded VitalPatch data on 1–21 days, summing up to a total of 1,297 days. Table 4 describes the demographics in each cohort. The patients were diagnosed on average 11.2 years before participation (SD 8.4, ranging from less than a year to 35 years, excluding 14 unknown time of diagnosis). All disease cohorts, excluding HD, included at least one participant unable to work (14 in total), while other participants worked full- or part-time, or were retired. Some IBD participants even worked several part-time jobs. While most participants were Caucasian, four participants were of Asian ethnicity and belonged to IMID group. One participant was African American and belonged to the healthy group.

**TABLE 4:** Demographics of study participants using VitalPatch.

Cohort group	Cohort	Sites	N	Female	Male	Years since diagnosis, mean (SD)	Years since diagnosis	Age, mean (SD)	Age range	BMI, mean (SD)
Healthy	Healthy	All	39	20	19	—	—	47.3 (16.3)	21–77	26.3 (4.9)
NDD	HD	G, K	13	7	6	4.8 (2.7)^a^	0–8^a^	44.2 (9.6)	30–60	26.3 (7.1)^b^
	PD	K	18	7	11	7.8 (5.9)	1–18^a^	62.3 (11.0)	37–80	24.3 (2.4)
IMID	IBD	E	18	9	9	12.9 (10.8)	1–35	36.7 (11.3)	22–55	24.7 (3.4)
	PSS	N	18	16	2	11.6 (5.4)	4–27	62.6 (13.1)	37–82	21.9 (10.3)
	RA	K, N	14	11	3	14.1 (9.4)	3–35	64.6 (12.2)	39–79	29.5 (7.9)
	SLE	K, N	16	16	0	16.7 (9.9)^a^	4–34^a^	48.3 (13.1)	31–80	23.1 (10.5)
*Total*	*7 cohorts*	*4 sites*	*136*	*86 (63.2%)*	*50 (36.8%)*	*11.2 (8.4)*	*0–35*	*51.6 (16.1)*	*21–82*	*25.2 (7.0)*

^a^ Four HD, patients, one PD, patient, and nine SLE, patients with unknown years since diagnosis.

^b^ Four HD, patients with unknown BMI.

**FIGURE 2:**
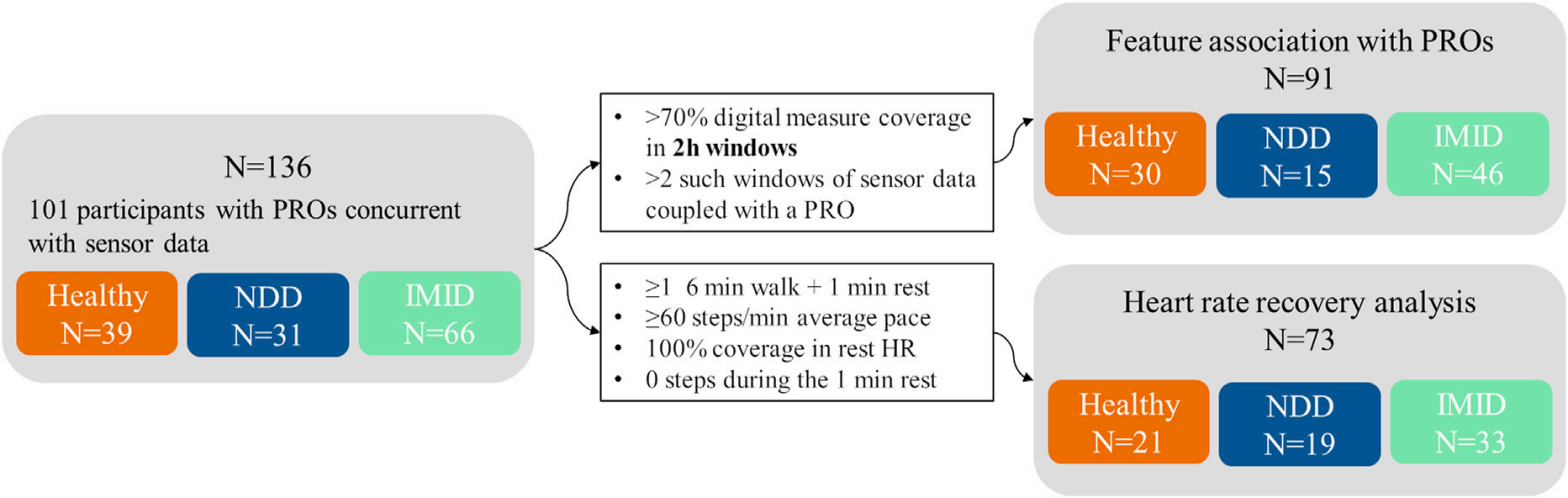
Participant flow diagram. The number of participants (N) in total and included in different analyses, presented with the inclusion criteria (black-bordered box).

A flow diagram illustrating different stages of analyses and their participant sample size is shown in Figure 2. A subset of 91 participants were applicable for the analysis of association between the digital measures and PROs. The concurrent measurements of digital measures and PRO data totalled 632 days, varying between 1 and 12 days per participant, and included 15 NDD patients (6 HD, 9 PD), 46 IMID patients (12 IBD, 13 PSS, 10 RA, 11 SLE), and 30 healthy controls.

All VitalPatch data were scanned for sequences applicable for heart rate recovery analysis. In total, 73 participants were included in the HRR analysis, comprising 19 NDD patients (9 HD, 10 PD), 33 IMID patients (11 IBD, 8 PSS, 8 RA, 6 SLE), and 21 healthy participants.

### 3.2 Data quality

In all VitalPatch data measured throughout the study, 2.3% of skin temperature data were range outliers, while only contextual outliers were identified for HR and RR (0.2% and 0.1%, respectively). For R-to-R intervals, less than 0.5% were outliers (0.1% invalid, 0.3% range and 0.1% contextual outliers). After outlier processing, the average daily coverage rates were 71.6% (16.3% SD) for HR, 71.7% (16.3% SD) for R-to-R interval, 70.9% (16.9% SD) for RR, and 65.5% (25.3% SD) for skin T. Moreover, the median daily coverage was 77% for skin T and about 78% for all other features. Hence, the sensor was typically worn for most of the day.

**FIGURE 3:**
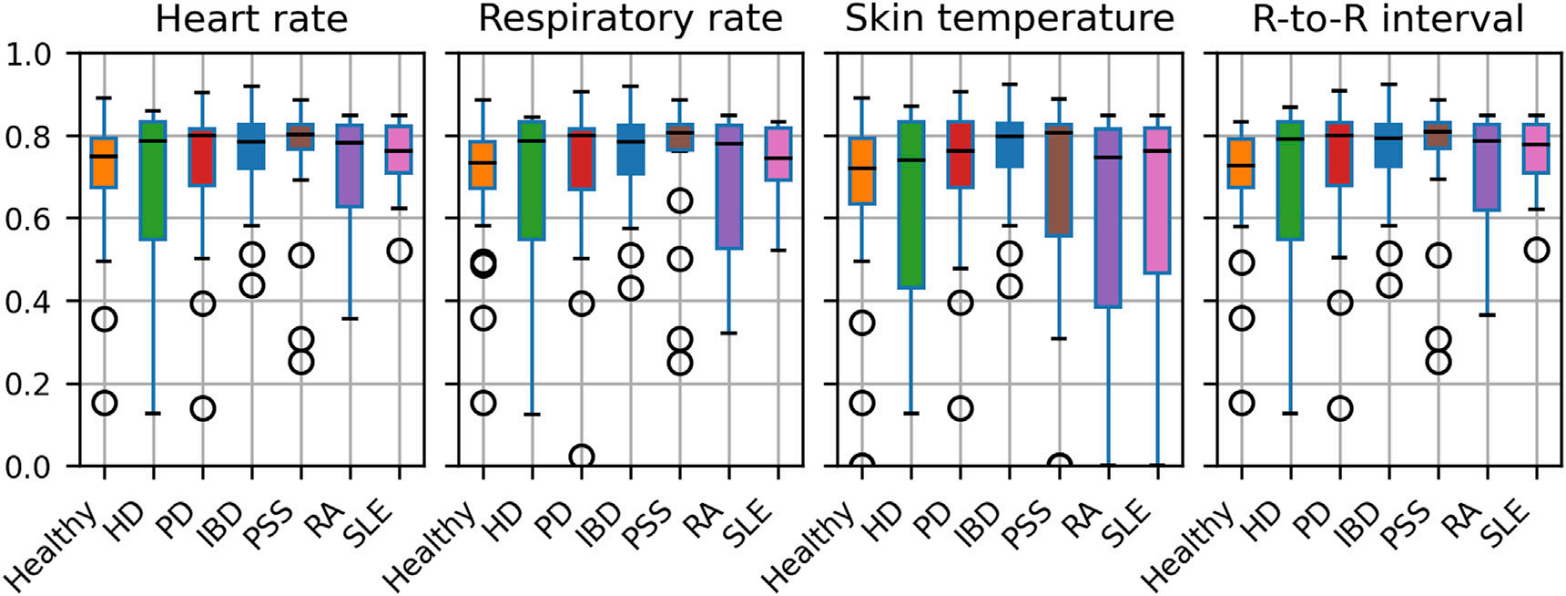
Average daily coverage of the cleaned digital measures across participants, presented by cohort.

Figure 3 illustrates the obtained coverage for each digital measure in each study cohort. While the smaller cohort groups (HD, RA) exhibit higher variation, the medians are comparable across cohorts. Notably, 11 participants stand out with zero coverage for skin T. However, further inspection revealed that these participants were recruited at the Newcastle site. The outliers could potentially indicate a need for improved device usage instructions at one site.

Table 5 presents the coverage of digital measures in the selected 2-h windows, analysed for association with PROs. Only skin temperature, which failed for 11 participants (including healthy, PSS, RA, and SLE participants), shows notable coverage differences across participant groups. The median coverage in the 2-h windows was 100% for all measures (10% percentile was 95.9% for skin temperature and above 99% for all other measures).

**TABLE 5:** Mean (with 95% confidence interval) feature coverage (%) in the 2-h windows preceding PRO responses, presented by participant group. Confidence intervals are based on the empirical rule (2*SD).

	Heart rate	R-to-R interval	Respiratory rate	Skin temperature
Healthy	99.3 [91.6, 100]	99.4 [91.7, 100]	98.9 [87.0, 100]	89.0 [27.7, 100]
NDD	99.4 [93.7, 100]	99.6 [94.0, 100]	99.4 [93.3, 100]	99.6 [94.1, 100]
IMID	99.3 [93.3, 100]	99.5 [93.7, 100]	98.9 [86.7, 100]	92.3 [40.6, 100]
Total	99.3 [92.8, 100]	99.5 [93.1, 100]	99.0 [87.6, 100]	92.5 [41.3, 100]

**FIGURE 4:**
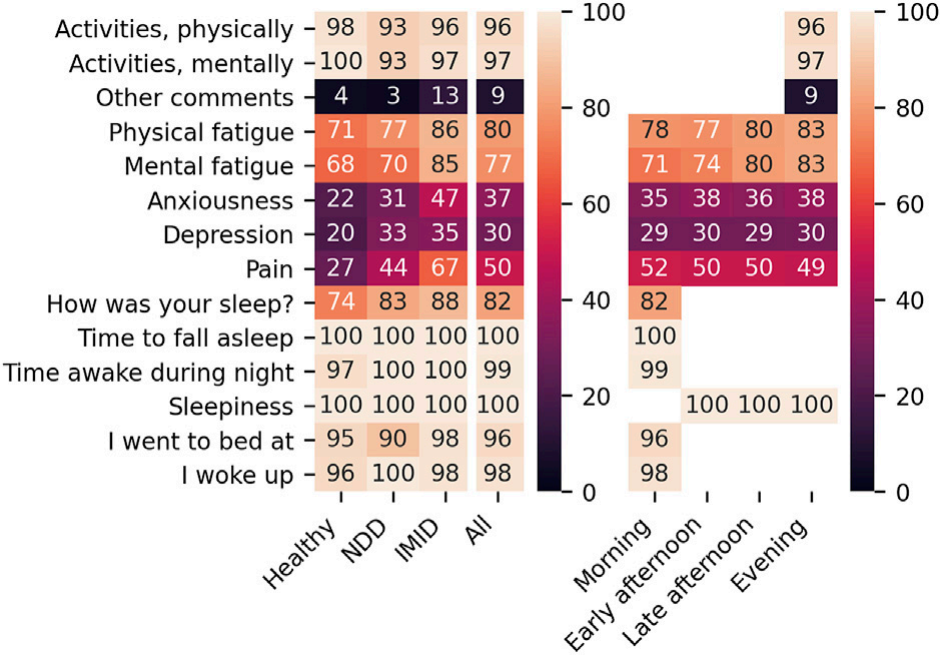
Coverage (%) of PROs per participant group (left) and questionnaire timing (right).

The coverage of PROs corresponding to the 2-h feature aggregates, analysed for association with digital measures, are presented in Figure 4. The analysis focuses on Likert item or drop-down menu PROs with overall coverage beyond 70%.

**FIGURE 5:**
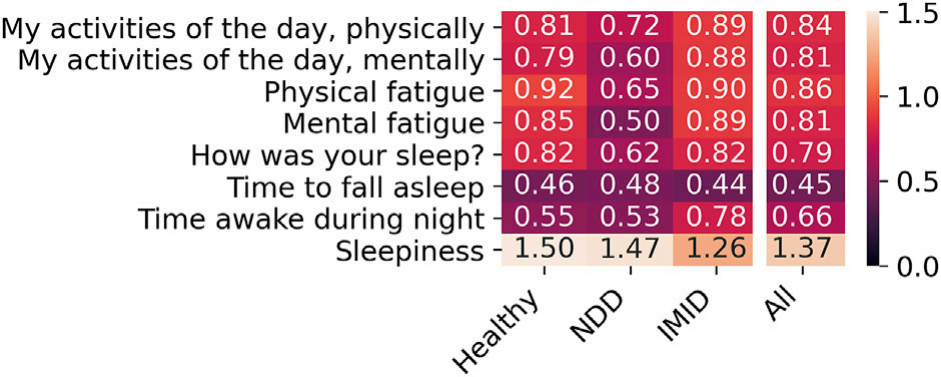
The SD of responses across participant groups. Here, the PROs correspond to the feature aggregates normalized with the participant-mean L5 window parameters.

Figure 5 presents SD captured in the PRO responses. The median number of distinct responses received from a participant was 2 for the activity and sleep detail questions, 3 for the fatigue questions, and 4 for the sleepiness question. The PRO response distributions were similar from participant group to another. Because the drop-down menu PROs (time to fall asleep and time awake during night) exhibit low variability, they are excluded from further analyses.

Self-reported sleep times were obtained for 244 nights concurrent with the VitalPatch data, allowing a comparison between reported sleep times and the extracted L5 periods. Overall, 68.4% of the L5 periods were entirely within the reported sleep time (82.0% started and 86.5% ended within the reported sleep time), and 86.9% were within a 30-min threshold of the reported sleep time (92.6% started and 94.3% ended within the reported sleep time). All L5 windows overlapped with the reported sleep times to some degree: in the case of least overlap, the L5 window started 3 h and 19 min before the reported sleep time. We note that the comparison only covers 54.6% of the total 447 extracted L5 periods.

### 3.3 L5 features in participant groups

**FIGURE 6:**
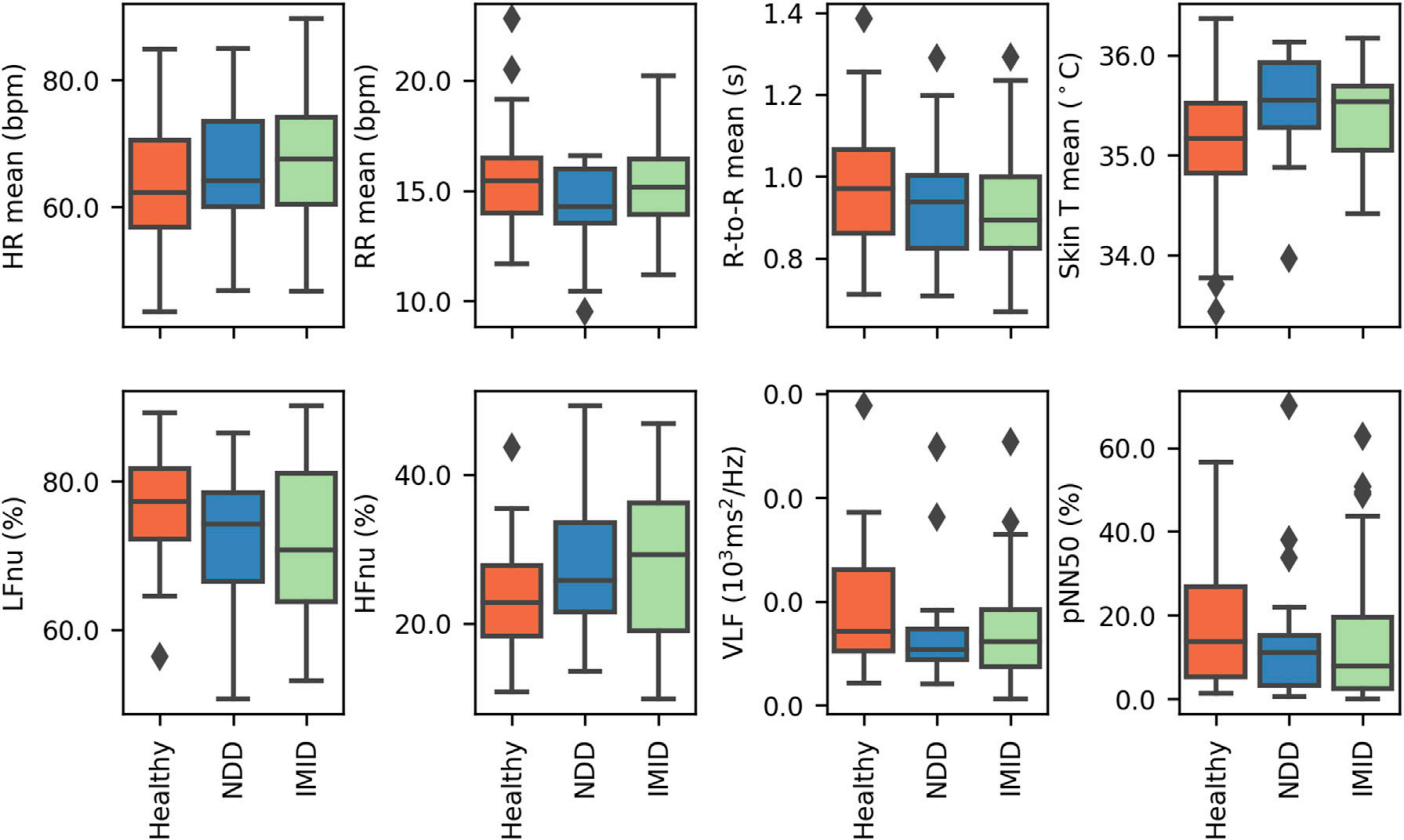
The participants’ average L5 parameter, presented by participant group, for (top) the mean signal values and (bottom) selected frequency domain HRV features. The figure covers 31 healthy participants, and 21 NDD and 49 IMID patients.

The mean resting time (L5) physiological measures for HR, RR, R-to-R interval, and skin T are compared across participant groups in Figure 6 (top). The average L5 mean HR observed for healthy participants was lower than that of either of the disease groups, and similarly mean R-to-R interval was higher. Additionally, a larger variety of L5 mean skin T was observed for the healthy group.

Selected L5 HRV parameters are similarly presented in Figure 6 (bottom). The frequency-domain features (LFnu, HFnu, and VLF) show some variations in the value distributions across groups. In accordance with the above-mentioned mean R-to-R interval distributions, pNN50 shows most R-to-R intervals exceeding 50 ms in the healthy group.

### 3.4 Feature aggregate association with PROs

**FIGURE 7:**
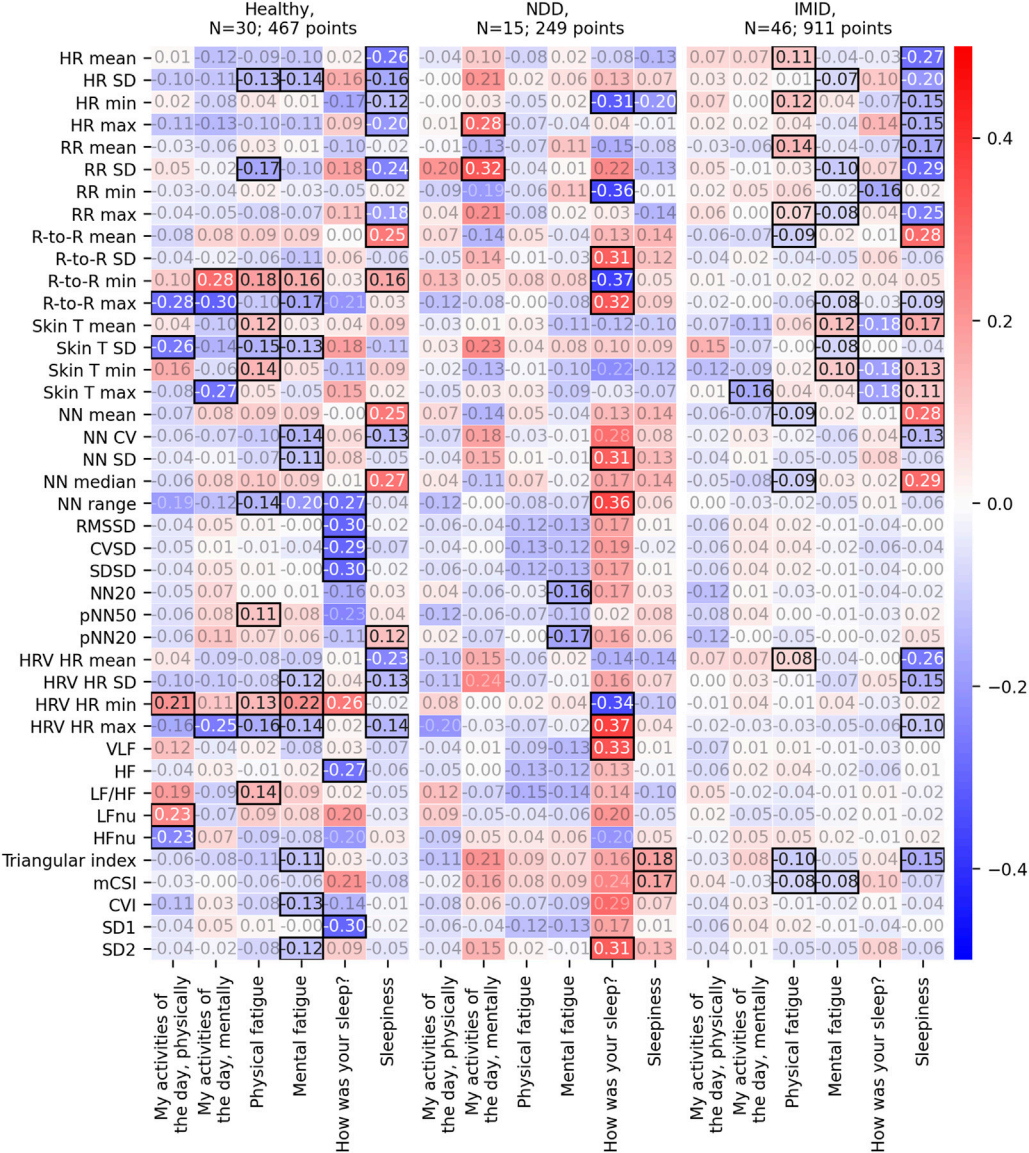
Repeated measures correlation r values between the 2-h feature aggregates and the corresponding PROs. Here, the 2-h feature aggregates have been normalized with the participant-mean L5 parameters. Statistically significant correlation results (*p*-value<0.05) are emphasized with black borders, other r values have faded annotation.

The association analysis between the 2-h aggregated features and the PROs comprised a total of 1,646 (476 for healthy, 253 for NDD, and 917 for IMID group) comparable instances collected from 91 participants. The analysis revealed statistically significant correlations between PROs and several feature aggregates. Figure 7 depicts the correlation r values for each participant group when the feature aggregates were normalized using the L5 participant-mean parameters. The corresponding *p*-values, degrees of freedom, and 95% confidence intervals are presented in Supplementary Figures S1–S3, respectively. For the healthy and IMID patients, the most pronounced correlations are close to ± 0.3, most of them for sleepiness PRO. NDD group shows most of the statistically significant correlations with sleep quality (|r| = 0.31–0.37).

**FIGURE 8:**
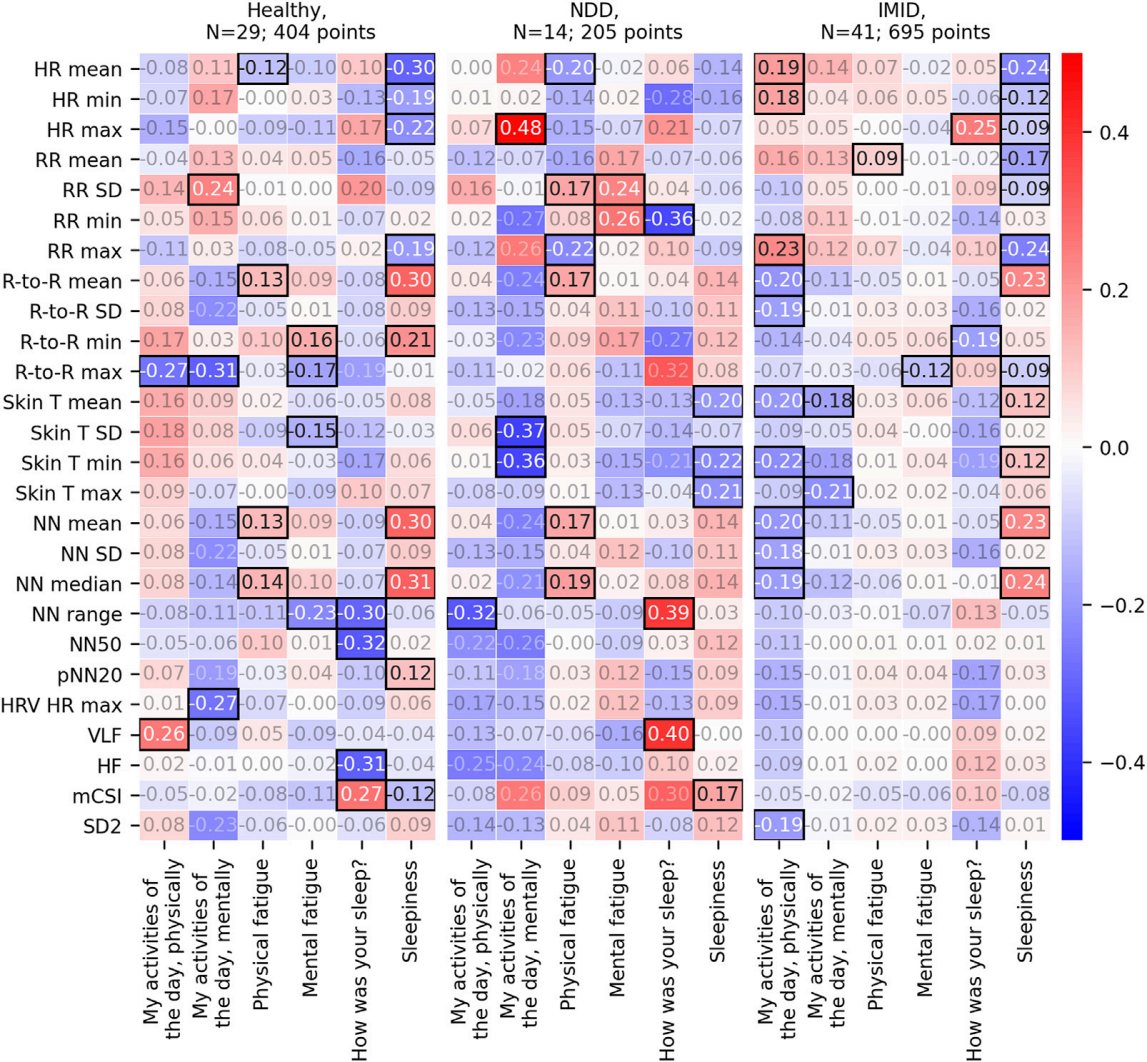
Repeated measures correlation r values between the 2-h feature aggregates and the corresponding PROs. Here, the 2-h feature aggregates have been normalized with the participants’ latest L5 window parameters. That is, as compared to Figure 7, the normalization parameters are not averaged over the full study periods. Statistically significant correlation results (*p*-value<0.05) are emphasized with black borders, other r values have faded annotation.

Figure 8 displays the correlation r to 2-h feature aggregates (see Supplementary Figures S4–S6 for the *p*-values, degrees of freedom, and 95% confidence intervals, respectively) normalized using the most recent previous L5 window parameters. It includes 1,319 (410, 209, and 700 for the healthy, NDD, and IMID group, respectively) PRO responses coupled with feature aggregates from a total of 84 participants. This is less than above because a normalization window with the set requirements was not always available. In this case, digital measure coverage shows significant correlation with mental daily activities. For the NDD group, the significant correlations are more spread over PROs. In the IMID group, features correlating with daily activity levels emerge.

### 3.5 Heart rate recovery

**FIGURE 9:**
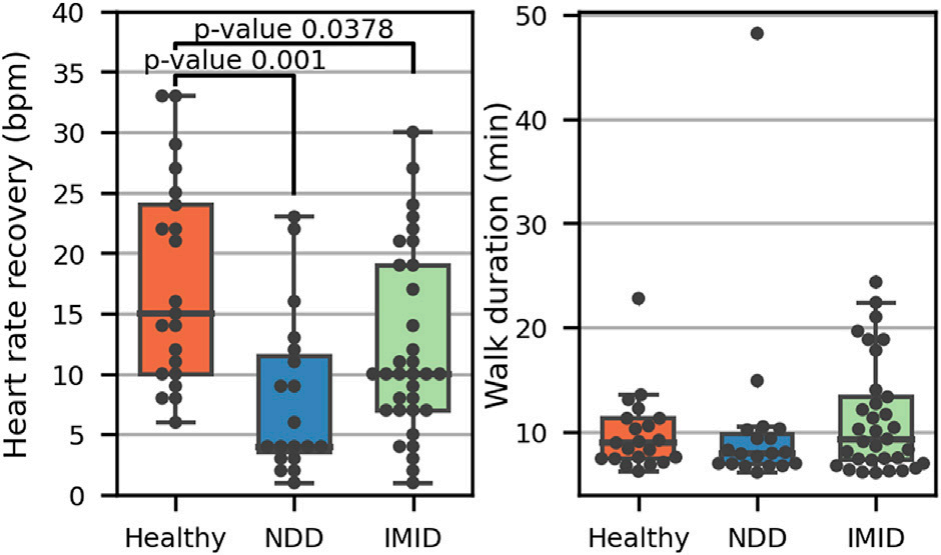
Heart rate recovery (left) in the three participant groups, including one representative value for 21 healthy participants, 18 NDD (9 HD, 10 PD), and 33 IMID patients (11 IBD, 8 PSS, 8 RA, 6 SLE). The adjusted *p*-values from post hoc analysis with Tukey’s method indicate the significance of the differences. The total duration of each accepted walk leading to a 1-min resting period is presented on the right.

The full 1,297 days of VitalPatch data were scanned for sequences applicable for heart rate recovery analysis. A total of 274 applicable HRR resting periods were identified, covering 73 distinct participants, as detailed in Table 6. Each participant (among the 73) had 1–16 applicable periods (3.8 on average). HRR by participant group is presented in Figure 9, with the total walk durations of the accepted walks. Only one representative HRR value (the highest) is depicted for each participant.

**TABLE 6:** Number of participants and sequences applicable for HRR analysis, and the median duration of walks leading to the inspected resting period.

Group	Cohort	N	Sequences for HRR analysis	Median walk duration (min)
Healthy	Healthy	21	91	9
NDD	HD	9	21	9
	PD	10	27	8
IMID	IBD	11	32	11
	PSS	8	45	11
	RA	8	36	9
	SLE	6	22	8
*Total*	*7 cohorts*	*73*	*274*	*9*

ANCOVA showed a significant difference with an F statistic of 5.68 (*p* < 0.006) in HRR between participant groups while adjusting for age and gender. The partial η^2^ implied a small effect, with 14% of the variance explained by the group (and 10% by age while the effect of gender was not significant). Post hoc analysis indicated that the healthy group differs significantly from both the NDD (T 3.95, *p* = 0.001) and IMID (T 2.51, *p* < 0.038) groups.

Among the 73 participants included in the HRR analysis, 65 had reported PROs during the full study period (3 participants among healthy and NDD patients and 2 among IMID patients had no response). The mean score for physical fatigue was 2.00 in healthy, 2.33 among NDD patients, and 2.39 in IMID patients. HRR’s relation to fatigue is explored further in the Supplementary Material S1 (see Supplementary Figures S7, S8). Significant HRR differences between high and low fatigue groups were observed only within the healthy participants.

## 4 Discussion

Fatigue and sleep disturbances reduce the quality of life and the activities of daily living. Digital measures collected with wearable devices could improve the objectivity and sensitivity of fatigue and sleep assessment, ultimately providing additional support for disease assessment and evaluation of new therapies. Wearable technologies could facilitate continuous monitoring outside the clinical setting without requiring active interaction from the patient. Moreover, digital measures in free-living settings may enable assessment that is more meaningful to the patient’s daily living. However, their potential for fatigue assessment have not been extensively studied, especially in the clinical context.

The results presented in this study suggest the feasibility of collecting reasonable quality physiological measures with a wearable biosensor on patients with chronic NDD and IMID diseases, as well as healthy controls. The median coverage was 77%–78% for all digital measures, with minimal variability across different cohorts. The coverage result implies high compliance to using the wearable biosensor. In contrast, only 91 among the 136 participants reported PROs at least three times during the study. Furthermore, in all the collected VitalPatch data, less than 0.5% of HR, RR, and R-to-R interval data and only 2.3% of skin temperature data needed to be cleaned out, indicating a sufficient data quality given the criteria used in this study.

To evaluate the association between the digital physiological measures and fatigue and sleep, we presented results of repeated measures correlation. We selected to evaluate the association between features aggregated over a 2-h window prior to a self-evaluation instance. Thus, the results represent the relationship between the 2-h physiological measures and the PRO at any time of the day. In the morning, the 2-h window may overlap with sleep. To account for the natural person-to-person variability in the digital measures, we further normalized the aggregated features with respect to the participant’s average parameters at rest, representing their typical resting state. The L5 windows representing rest were identified utilizing the activity measures available from the same wearable sensor. The L5 periods were reasonably aligned with the self-reported sleep times. While no major differences were observed in the participant-mean L5 parameters themselves across participant groups, some expected variations appeared. For instance, we observed lower mean HR and higher R-to-R intervals for the healthy, which is consistent with the presumption that increased fatigue is associated with reduced HRV (Escorihuela et al., 2020). Interestingly, the NDD group showed higher skin temperatures than others, with less variation, too. Although the group is small, this observation is in line with study by Eggenberger et al. (2021) where they found that cognitively healthy adults have lower skin temperatures than those with mild cognitive impairment. It is noted that the skin temperature may be affected by ambient temperatures.

The statistically significant correlations between the 2-h feature aggregates and the PROs varied from participant group to another. For NDD patients, most of the significant correlations associated with sleep quality. For the IMID patients, most correlations were found for sleepiness, whereas a reasonable number of correlations were also identified for both physical and mental fatigue. The same is true for the healthy participants, although there is some variance in the specific digital measures that correlate with the PROs.

We also proposed an alternative method for feature normalization, which uses the latest L5 parameters instead of the averaged ones. Using this method revealed correlations for physical and mental fatigue also in the NDD patient group. In the IMID group, significant correlations with the physical activities of the day emerged. This normalization approach may be better able to account for shifts in the daily baseline.

Inspecting the individual feature aggregates in Figures 7, 8 further imply the relevance of the digital measures. HR is relevantly associated with sleepiness, both in the healthy group and IMID patients. Interestingly, this association is not seen in the NDD group, suggesting that neurodegeneration breaks this association, e.g., by affecting the central autonomic nuclei and/or pathways. Significant associations between skin T and the dependent variables in the healthy and the IMID patients, but not in NDD patients in Figure 7, suggest a similar mechanism. These observations may be related to the circadian rhythm abnormalities in NDD patients reported in previous studies (Hood and Amir, 2017). The LF/HF ratio, which in controlled settings reflects the ratio between sympathetic nervous system and parasympathetic nervous system activity, was associated with daytime symptoms in the healthy, but not in the NDD and IMID patients, suggesting an affection of this balance in NDD and IMID in the daytime. It is also noteworthy that in NDD most of the significant results occur between the dependent variables and sleep quality, and in IMID between dependent variables and (daytime) sleepiness, which speaks for different mechanisms of vegetative control between the different types of diseases. Conversely, it is also interesting to observe that sleepiness and mental/physical fatigue obviously represent different concepts and mechanisms, since the distribution of the significances for the respective variables is very different. A detailed analysis of the clinical relevance of the findings is, however, out of the scope of this work and will be left for future research. The clinical implications of free-living heart rate variability details may require further examination (Hayano and Yuda, 2019, Hayano and Yuda, 2021). Since wearable devices often utilize a lower sampling frequency to reduce power consumption and prolong the battery life, careful consideration on the sampling rate should be made during experiment planning. Although prior work has demonstrated reliability and clinical utility of heart rate variability measures quantified from a sampling frequency of 125 Hz (Ellis et al., 2015; Nallathambi et al., 2020; Hirten et al., 2021; Lee et al., 2022), very low variability in R-to-R interval, such as those observed in heart failure patients, may require higher sampling frequencies for sufficient temporal resolution (Kuusela, 2013).

We note that most of the correlations are modest, and a larger group especially of NDD patients is required to validate the presented findings. More advanced features beyond the 2-h statistical aggregators and classical HRV features should be studied in the future to capture more complicated temporal patterns. Additionally, although repeated measure correlation was selected to account for participant-to-participant differences in PRO reporting, the subjectivity and limited sensitivity of the PROs could limit the possibilities to detect associations.

The PRO-association analysis was complemented by an explorative analysis of 1-min HRR during rest, after periods of sustained activity. We discovered that the NDD and IMID patients showed significantly (*p* = 0.001 and *p* = 0.0378, respectively) lower HRR values as compared to the healthy controls. This finding is consistent with previous research indicating deteriorated HRR in the HD, PD, IBD, RA, and SLE cohorts as compared to healthy controls after an exercise test either on a treadmill or on a cycle ergometer (Dogdu et al., 2010; Sarli et al., 2016; Bienias et al., 2017; Pecąnha et al., 2018; Roberson et al., 2018; Steventon et al., 2018). The presented result suggests that the difference may also be observed in the context of daily walking activities using wearable technology in free living participants. While the 6-min walk test in controlled settings has been previously established as a valid test beside the more intense treadmill exercise, our results suggest that useful information can also be extracted from at-home continuous physiological measurements (Roberts et al., 2006). On the other hand, NDD are associated with disruption to blood flow, hypertension, and reduction in cerebral blood flow (Youwakim and Girouard, 2021). These factors may contribute to the variation in the HRR results in comparison to the healthy participants. For SLE, HRR deterioration has been suggested to associate with disease severity (Bienias et al., 2017). More research is required to assess the connection of HRR monitored by wearables to disease severity in the NDD and IMID patients.

The study did not show any significant association between HRR and fatigue in the patient groups, although on average the NDD and IMID patients reported higher fatigue than the healthy participants. However, the participant group correlated significantly to HRR and may act as a confounder whose effect dominates over that of fatigue. In the healthy group, in contrast, a significant difference in HRR was observed between high and low fatigue groups. Furthermore, because of the subjective differences in self-assessment of fatigue, the association between HRR in free-living settings and fatigue should be studied with repeated measures on a subject level, in a study covering a longer study period. A notably longer study period could also enable more advanced analysis, like evaluating the sensitivity of HRR to within-subject changes in fatigue.

Given the multifactorial nature of fatigue, future work will combine physiological measures studied here with multiple sensing modalities. For instance, acceleration signals could be utilized to investigate physiological responses in the context of specific activities, or physiological measures could be combined with the observed sleep stages to further investigate connections with sleep. The IDEA-FAST consortium intends to validate findings of this pilot study using multiple sensing modalities in a larger cohort of patient and healthy participants (*N* = 2000) and over a longer study period. The large-scale nature of this future study will enable further investigation on the sensitivity of HRR and other digital measures to changes in fatigue and sleep.

## Data Availability

The datasets presented in this article are not readily available because of the sensitive nature of the data. The study data will be made available upon request for validation purposes, subject to the signing of a suitable data sharing agreement. Validation can only take place on the IDEA-FAST data platform which supports all necessary statistical software tools. Requests to access the datasets should be directed to https://idea-fast.eu/contact/.
