# Supplementary material for: Assessing fatigue and sleep in chronic diseases using physiological signals from wearables: A pilot study
Source: Front Physiol. 2022 Nov 14;13:968185. doi: 10.3389/fphys.2022.968185 (PMC9702812; doi:10.3389/fphys.2022.968185)
Supplement: Supplementary file 1 [file DataSheet1.docx]

Supplementary Material

# Supplementary: Feature Aggregate Association with PROs


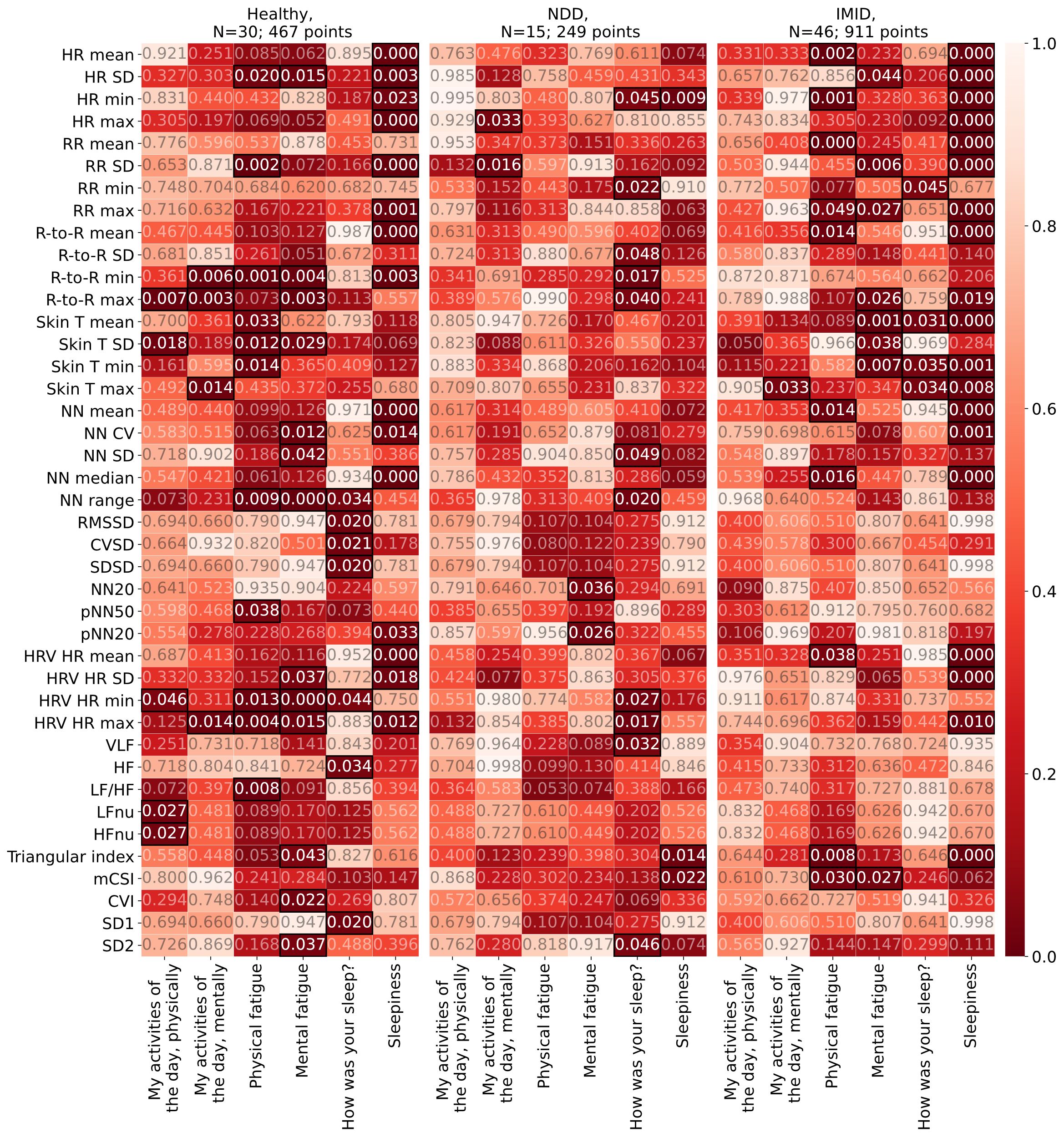


Supplementary Figure 1 Repeated measures correlation p-values between the 2h feature aggregates and patient reported outcomes (PROs). Here, the 2h feature aggregates have been normalized with the subject mean L5 parameters. P-values indicating statistically significant correlation (p-value<0.05) are highlighted with black borders and displayed with darker colour, whereas other p-values have faded annotation.


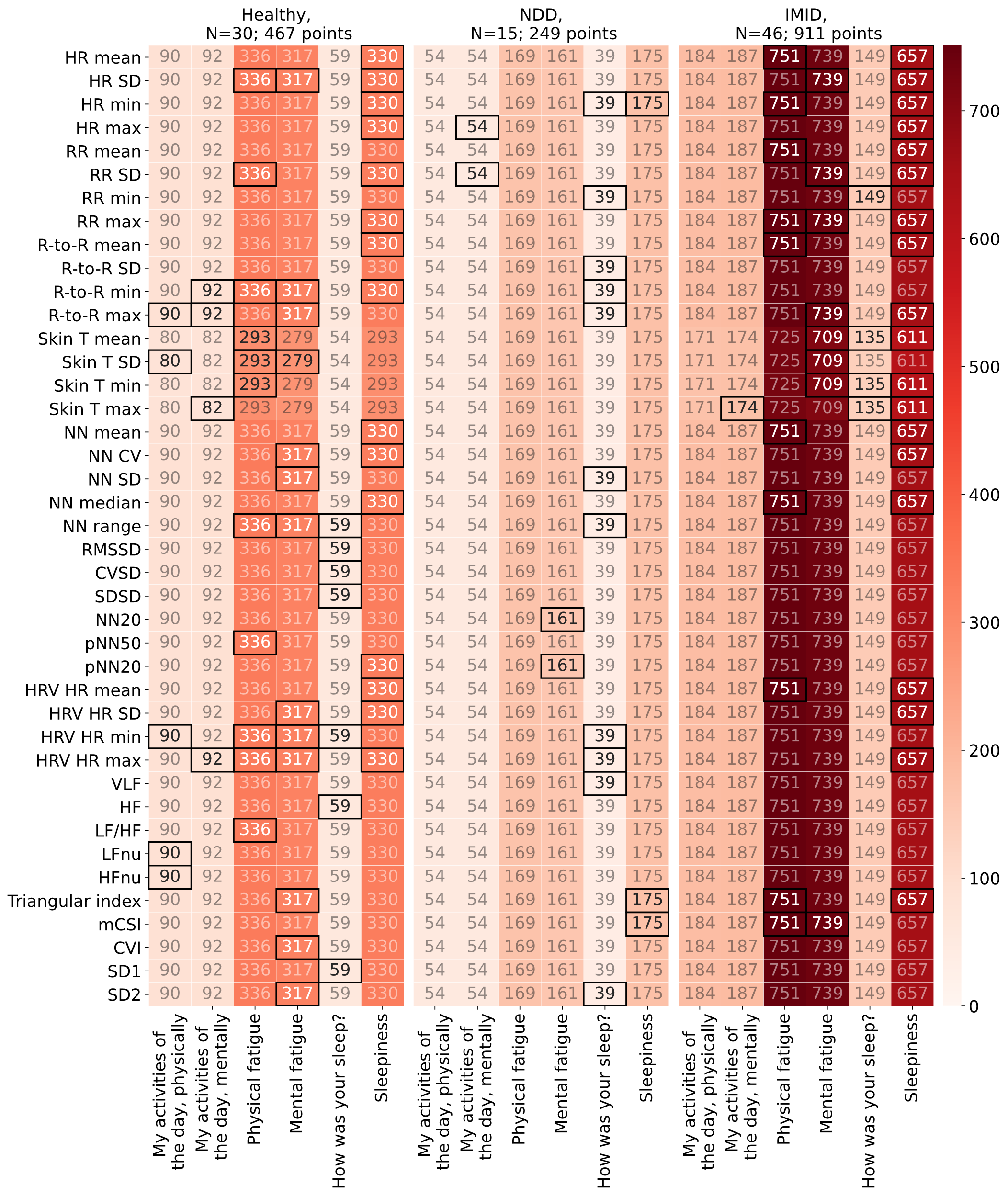


Supplementary Figure 2 The degrees of freedom for the repeated measures correlation between the 2h feature aggregates and patient reported outcomes (PROs), using the subject mean L5 parameters for normalization. Cells corresponding to significant correlations are highlighted with black borders.


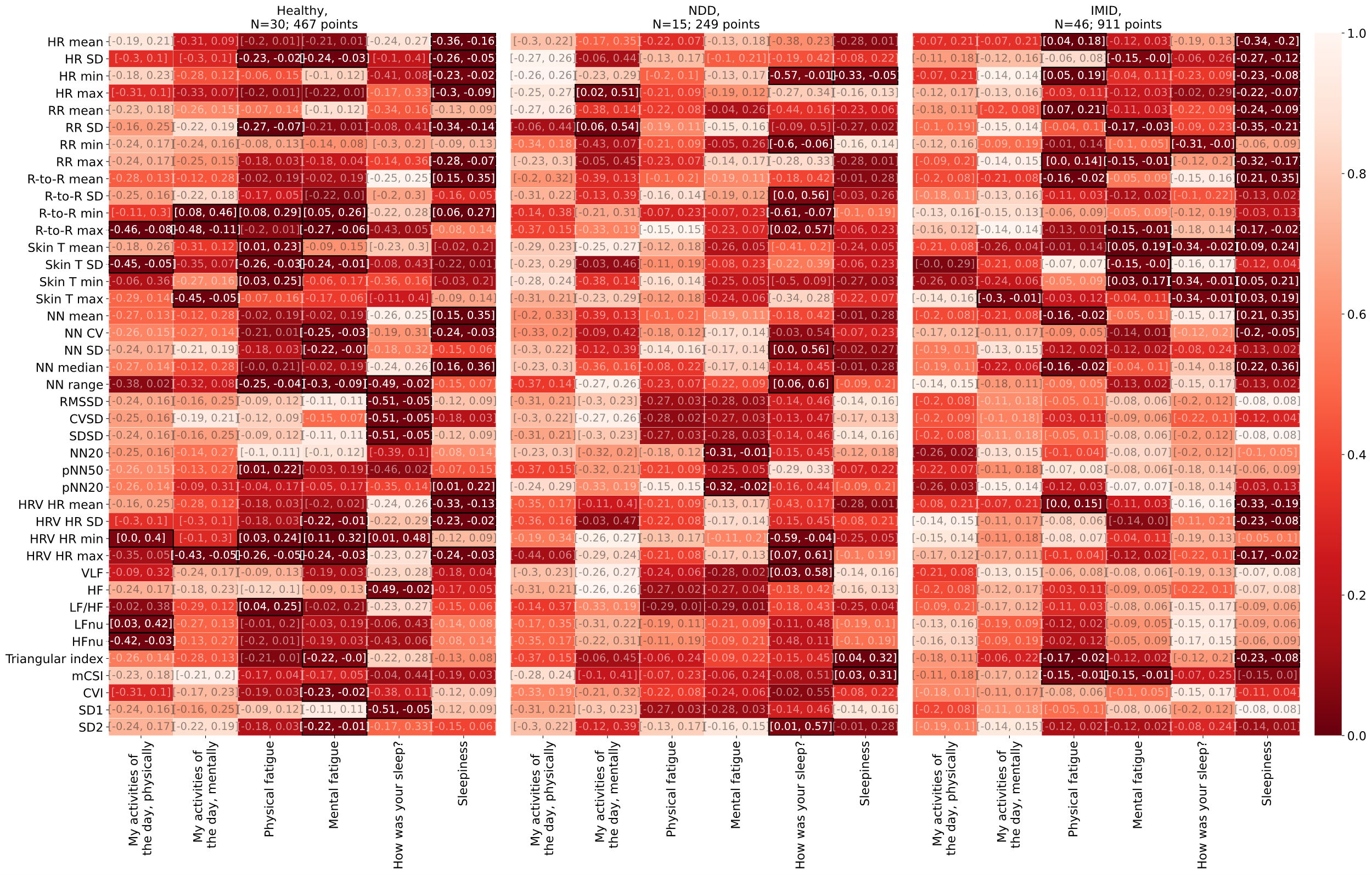


Supplementary Figure 3 The 95% confidence intervals for the repeated measures correlation between the 2h feature aggregates and patient reported outcomes (PROs), using the subject mean L5 parameters for normalization. Colors as in Supplementary Figure 1.


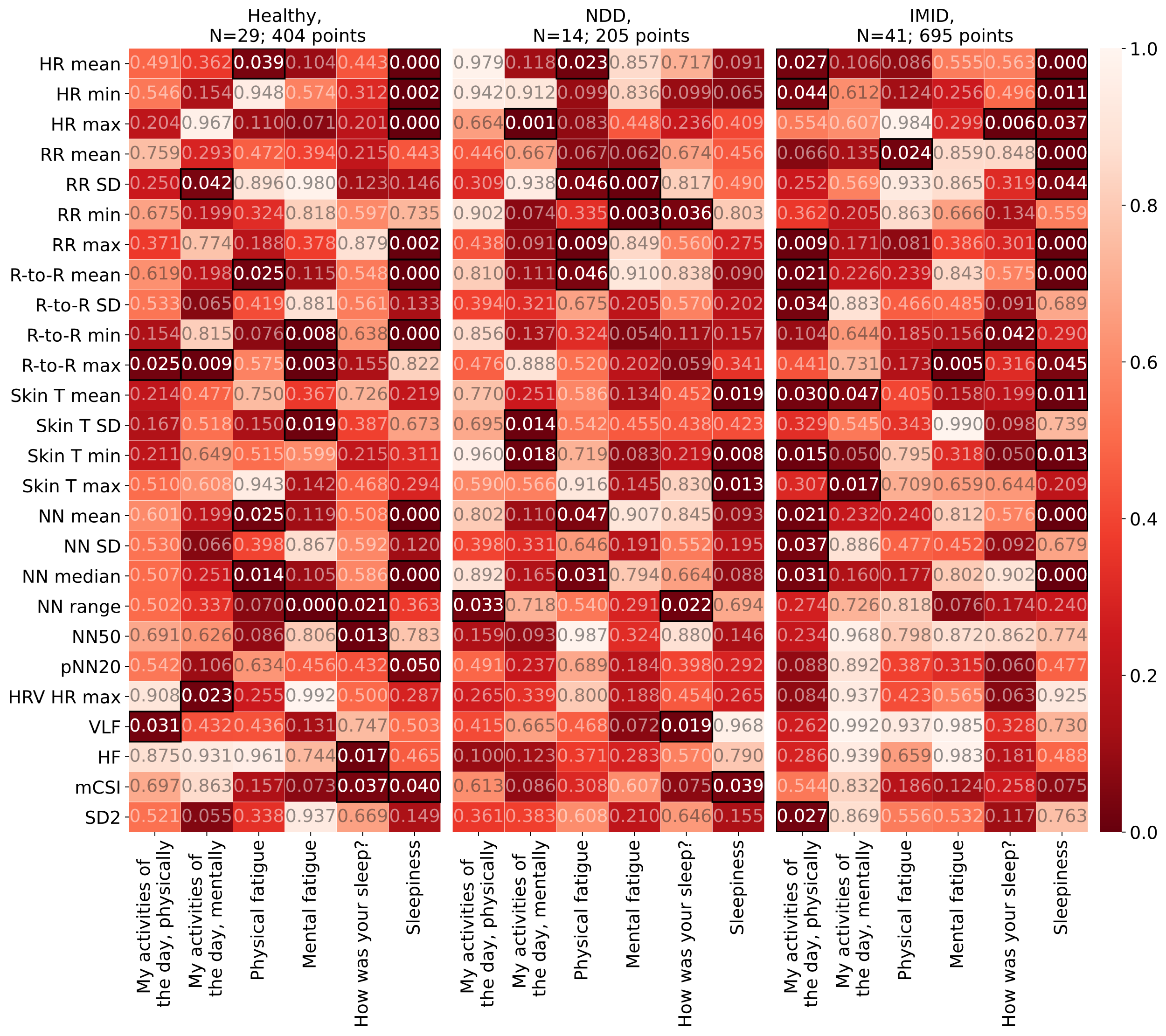


Supplementary Figure 4 Repeated measures correlation p-values between the 2h feature aggregates and PROs. Here, the 2h feature aggregates have been normalized with the subject’s latest L5 window parameters. P-values indicating statistically significant correlation (p-value<0.05) are highlighted with black borders and are displayed with darker colour, whereas other p-values have faded annotation.


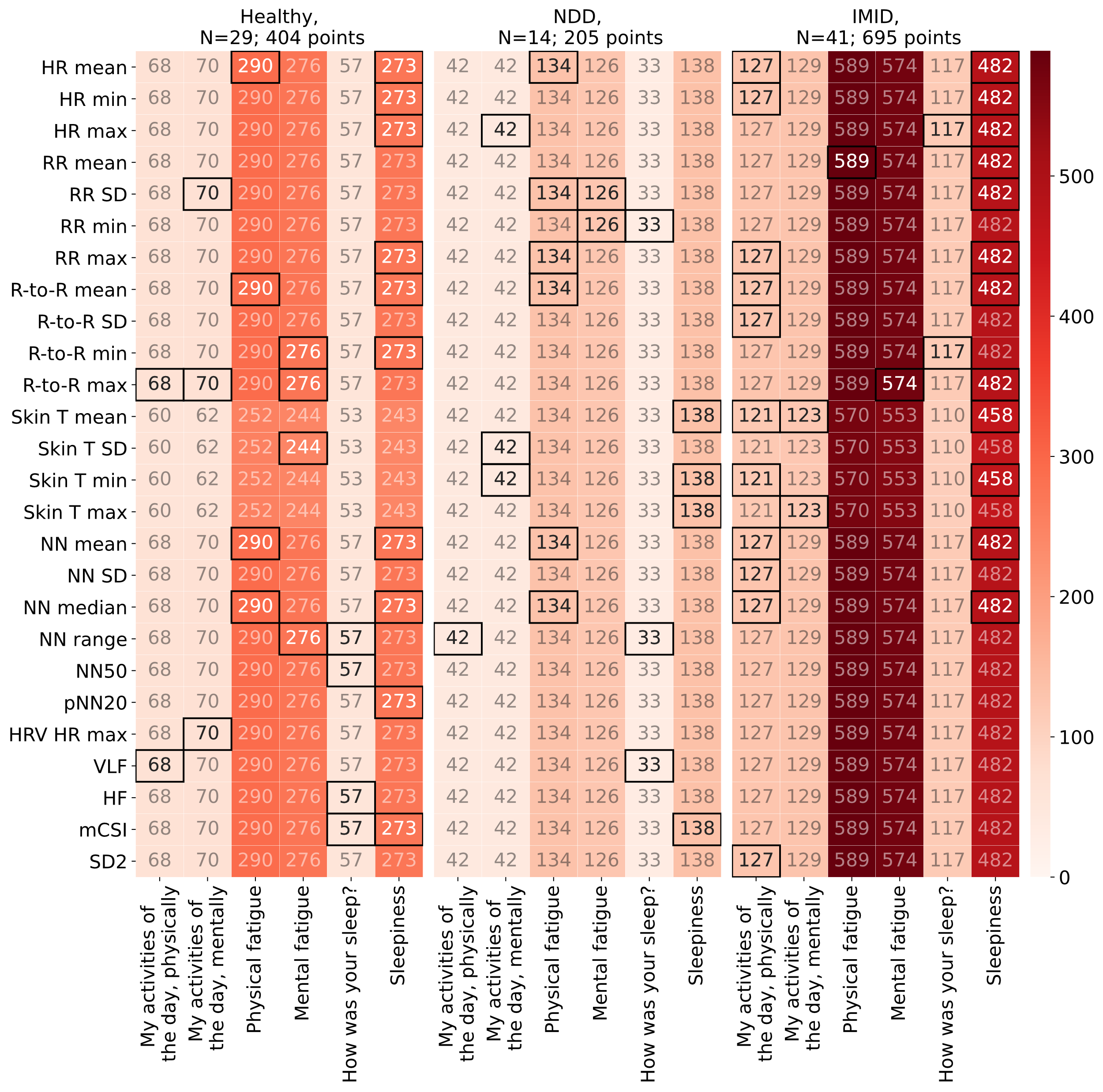


Supplementary Figure 5 The degrees of freedom for the repeated measures correlation between the 2h feature aggregates and patient reported outcomes (PROs), using the subject’s latest L5 parameters for normalization. Cells corresponding to significant correlations are highlighted with black borders.


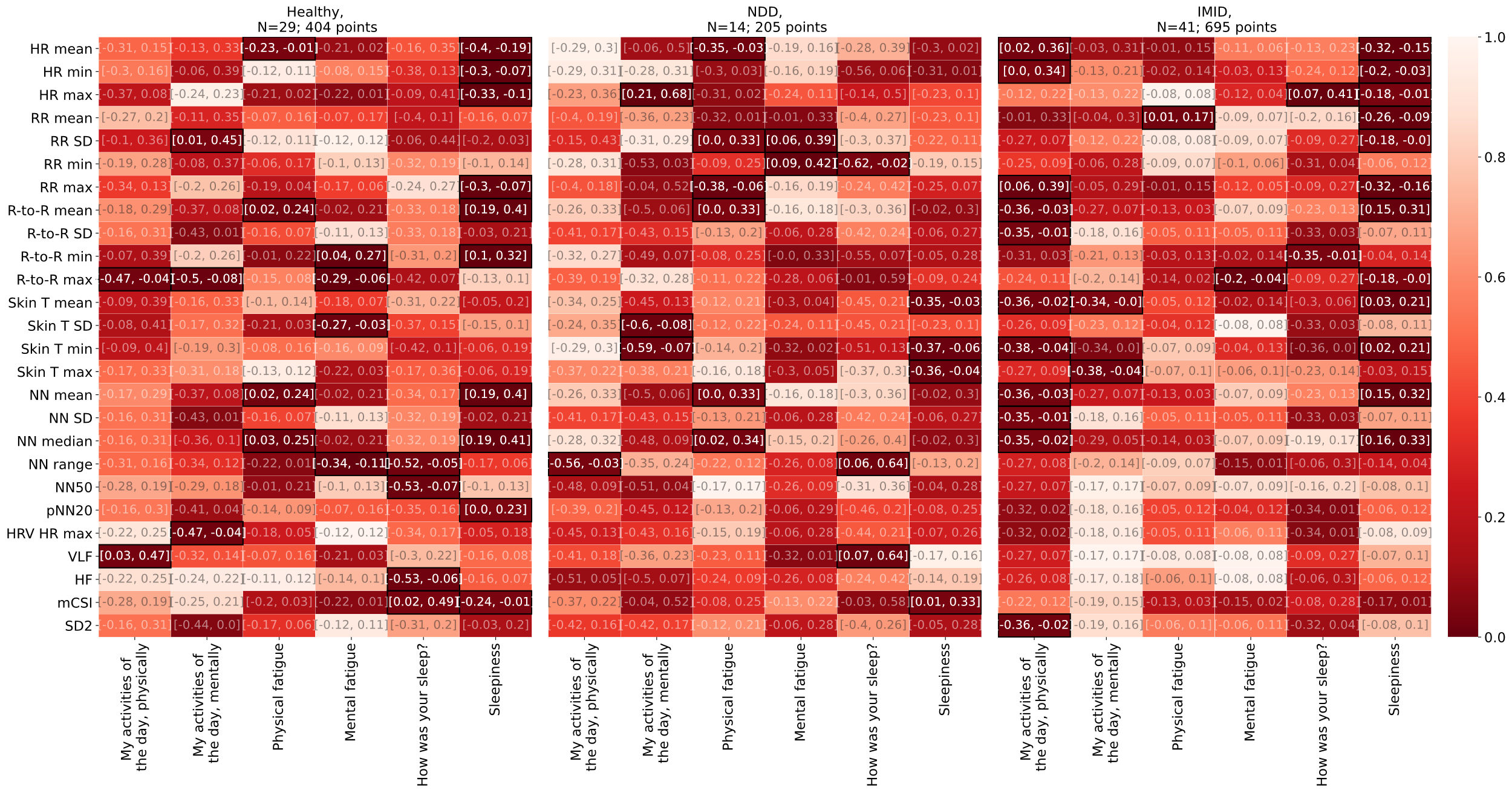


Supplementary Figure 6 The 95% confidence intervals for the repeated measures correlation between the 2h feature aggregates and patient reported outcomes (PROs), using the subject’s latest L5 parameters for normalization. Colors as in Supplementary Figure 4.

# Supplementary: Heart Rate Recovery Association with Fatigue

The median of participant-averaged fatigue was 1.98 for physical fatigue and 2.05 for mental fatigue, among all study participants. On the original integer scale, they correspond to 2.0. To investigate possible differences in heart rate recovery (HRR) among high and low fatigue groups, each participant group (healthy, NDD, IMID) were divided into low fatigue (average fatigue score up to 2.0) and high fatigue (average fatigue score above 2.0) groups. The two fatigue types were studied independently. A total of 65 participants eligible for the HRR analysis had also reported fatigue levels, while responses were missing from three healthy participants, three NDD patients, and two IMID patients. A non-parametric, two-sided Kolmogorov-Smirnov (KS) test was used to test for statistical difference between the two fatigue groups.

The results for physical fatigue are shown in Supplementary Figure 7 and for mental fatigue in Supplementary Figure 8. For both types of fatigue, the KS test showed significant difference between the high and low fatigue groups among healthy participants, with KS 0.77 (p-value 0.01) for physical fatigue and KS 0.68 (p-value 0.02) for mental fatigue. However, no significant differences were observed for the NDD patients (KS 0.50, p-value 0.28 for physical fatigue and KS 0.14, p-value 1.00 for mental fatigue) or the IMID patients (KS 0.18, p-value 0.92 for physical fatigue and KS 0.24, p-value 0.70 for mental fatigue).


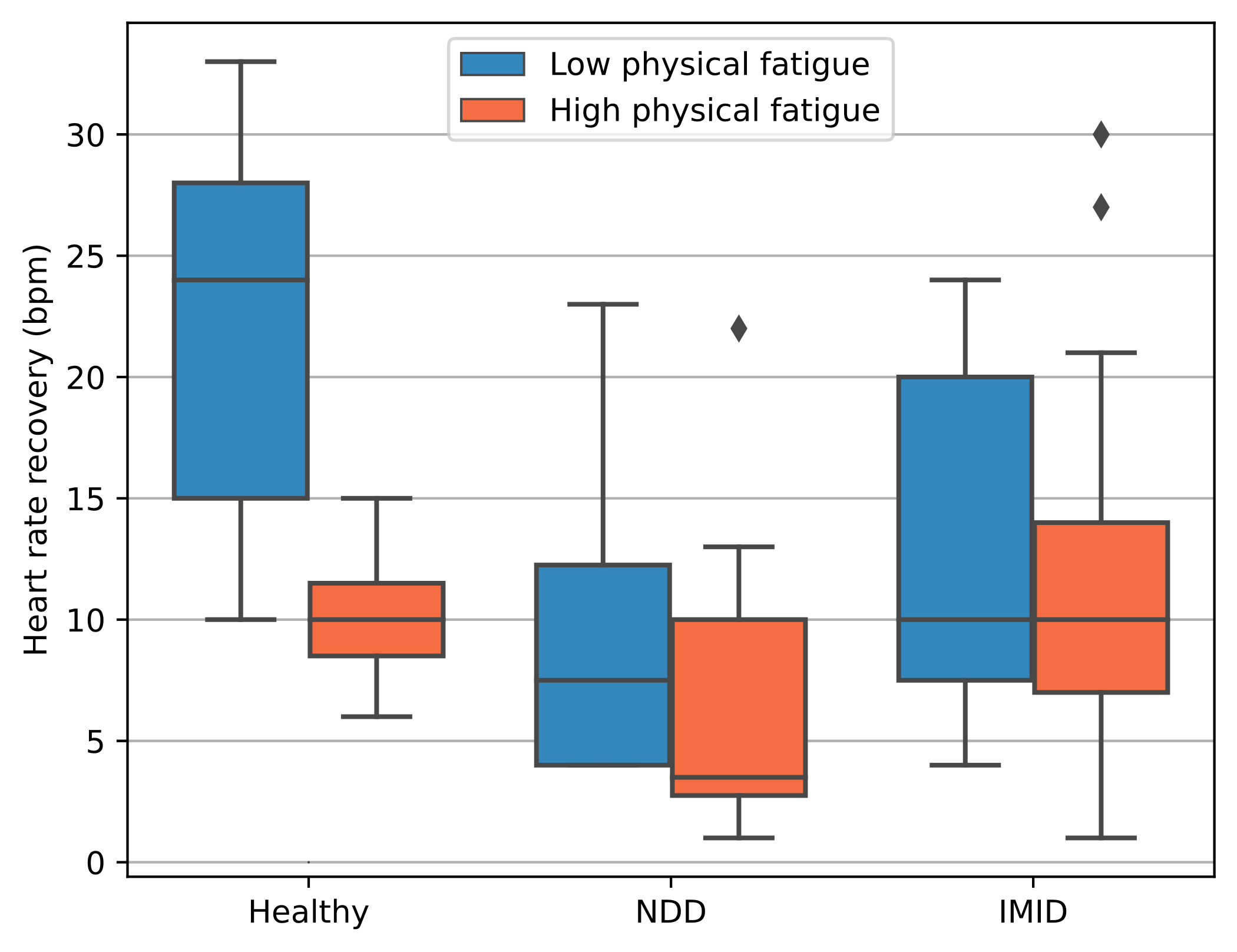


Supplementary Figure 7 Heart rate recovery in participants with low (≤2.0, blue) and high (>2.0, red) mean reported physical fatigue, presented by participant group. The healthy group comprised 7 high and 11 low fatigue participants, the NDD group 8 high and 8 low fatigue participants, and the IMID group 17 high and 14 low fatigue participants.


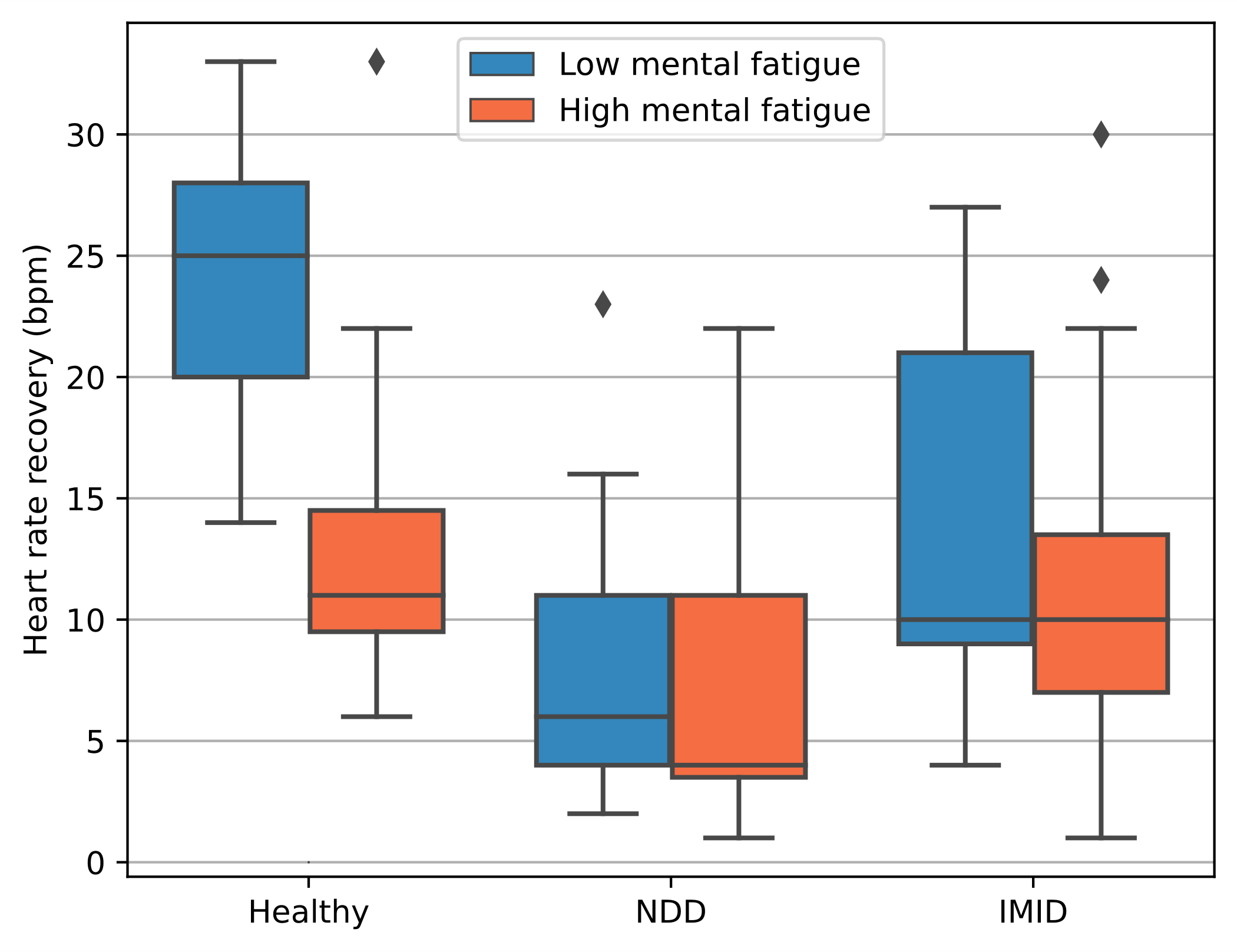


Supplementary Figure 8 Heart rate recovery in participants with low (≤2.0, blue) and high (>2.0, red) mean reported mental fatigue, presented by participant group. The healthy group comprised 11 high and 7 low fatigue participants, the NDD group 7 high and 9 low fatigue participants, and the IMID group 18 high and 13 low fatigue participants.
